# Curcumin’s mechanism of action against ischemic stroke: A network pharmacology and molecular dynamics study

**DOI:** 10.1371/journal.pone.0280112

**Published:** 2023-01-04

**Authors:** Yangyang Wang, Guoxiu Zu, Ying Yu, Jiqin Tang, Tao Han, Chengdong Zhang

**Affiliations:** 1 College of Rehabilitation Medicine, Weifang Medical University, Weifang, China; 2 Department of Traditional Chinese Medicine, Shandong University of Traditional Chinese, Jinan, China; 3 Innovative Institute of Chinese Medicine and Pharmacy, Shandong University of Traditional Chinese Medicine, Jinan, Shandong, China; Maulana Azad Medical College, INDIA

## Abstract

Ischemic stroke (IS) is one of the major global causes of death and disability. Because blood clots block the neural arteries provoking ischemia and hypoxia in the brain tissue, IS results in irreversible neurological damage. Available IS treatments are currently limited. Curcumin has gained attention for many beneficial effects after IS, including neuroprotective and anti-inflammatory; however, its precise mechanism of action should be further explored. With network pharmacology, molecular docking, and molecular dynamics (MD), this study aimed to comprehensively and systematically investigate the potential targets and molecular mechanisms of curcumin on IS. We screened 1096 IS-related genes, 234 potential targets of curcumin, and 97 intersection targets. KEGG and GO enrichment analyses were performed on these intersecting targets. The findings showed that the treatment of IS using curcumin is via influencing 177 potential signaling pathways (AGE-RAGE signaling pathway, p53 signaling pathway, necroptosis, etc.) and numerous biological processes (the regulation of neuronal death, inflammatory response, etc.), and the AGE-RAGE signaling pathway had the largest degree of enrichment, indicating that it may be the core pathway. We also constructed a protein–protein interaction network and a component–target–pathway network using network pharmacology. From these, five key targets were screened: NFKB1, TP53, AKT1, STAT3, and TNF. To predict the binding conformation and intermolecular affinities of the key targets and compounds, molecular docking was used, whose results indicated that curcumin exhibited strong binding activity to the key targets. Moreover, 100 ns MD simulations further confirmed the docking findings and showed that the curcumin–protein complex could be in a stable state. In conclusion, curcumin affects multiple targets and pathways to inhibit various important pathogenic mechanisms of IS, including oxidative stress, neuronal death, and inflammatory responses. This study offers fresh perspectives on the transformation of curcumin to clinical settings and the development of IS therapeutic agents.

## Introduction

Stroke has been identified as a global cerebrovascular disease with increased incidence in younger individuals and high rates of disability and mortality [1]. Stroke can be roughly divided into ischemic stroke (IS) and hemorrhagic stroke. The American Heart Association reports that IS accounts for 87% of total stroke patients [2]. IS is often caused by cerebral artery blockage due to cerebral thrombosis or embolism, resulting in ischemic lesions in the arterial blood supply area of the brain and sensory impairment of patients’ limbs [3]. Thrombolytic therapy is currently the main treatment for IS, but it has some limitations [4]. Cerebral ischemia-reperfusion following thrombolytic therapy may trigger inflammatory responses, which can further lead to secondary brain injury such as neuronal necrosis and brain tissue edema [5]. The limited time window for thrombolytic therapy reduces the number of patients who can receive effective treatment [6]. Therefore, identifying new drugs or chemical components to treat IS has become a current focus in the field.

Curcumin, a food-derived chemical, is a plant polyphenol extracted from turmeric rhizome [7]. Modern medical research has shown that curcumin has various pharmacological properties, including being anti-inflammatory, antioxidant, platelet inhibition, and apoptosis regulation [8–10]. At present, several studies have indicated that curcumin can protect the brain tissue and ameliorate nerve injury, thereby making it a promising IS treatment [11]. However, the potential targets and mechanisms of curcumin in IS treatment are yet to be determined. Network pharmacology is an emerging pharmacological research method that integrates bioinformatics and traditional pharmacology knowledge. It can be used to identify the targets of candidate treatments, as well as the functions and mechanisms of bioactive ingredients in disease treatment [12].

**Fig 1:**
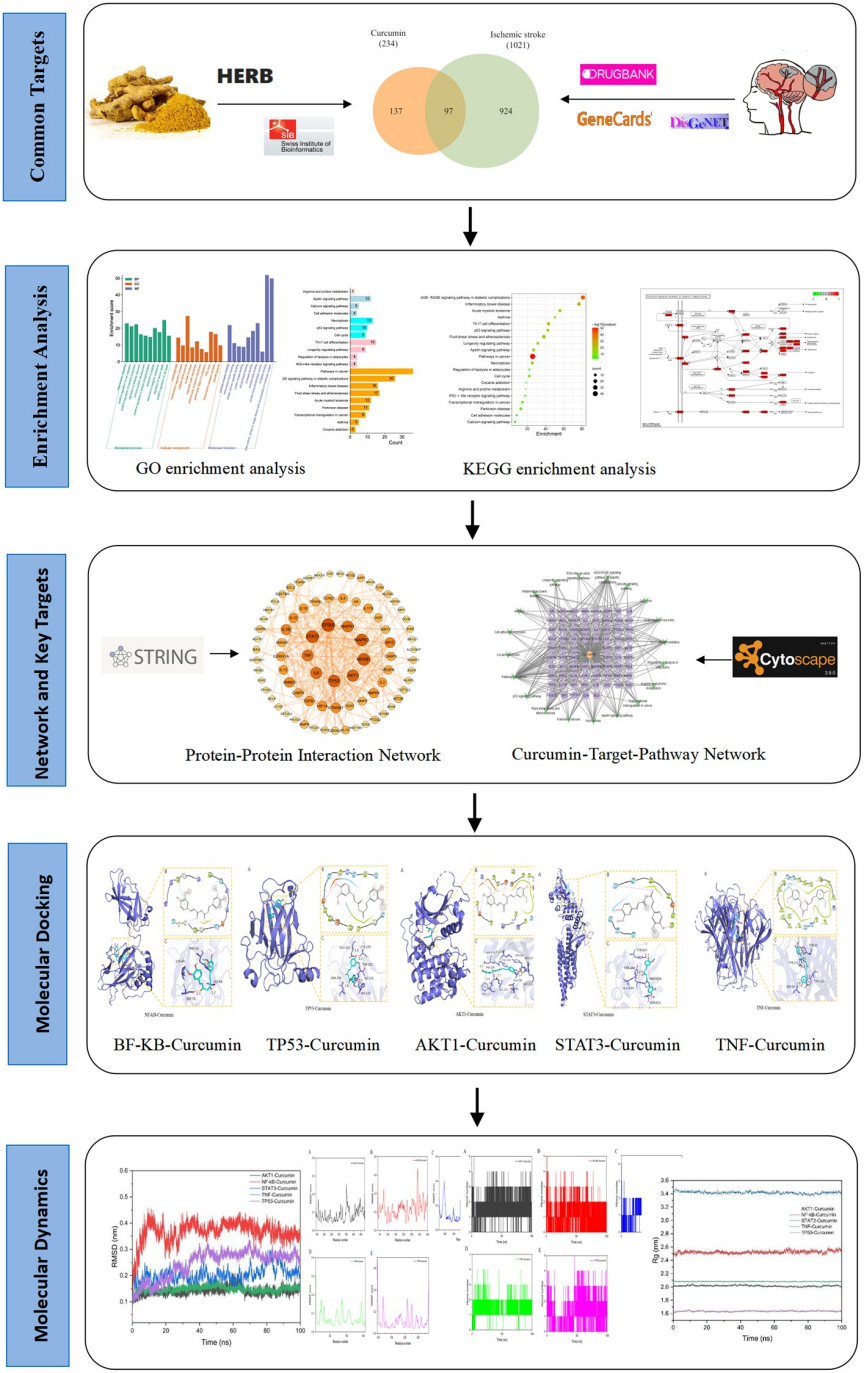
Flow chart of network pharmacology.

Given that curcumin is a potential therapeutic candidate for IS, this study used network pharmacology to comprehensively and systematically explore the potential targets and intricate mechanisms of how curcumin exerts therapeutic benefits. Additionally, we run molecular docking and molecular dynamic (MD) simulations to verify the stability and affinities of key targets and compound binding. Our study has crucial implications on how curcumin is applied clinically and how well we understand the fundamentals of stroke treatments. The flow chart of this network pharmacology study is shown in Fig 1.

## Materials and methods

### Curcumin target protein prediction

The protein targets for curcumin were screened in the HERB database (http://herb.ac.cn/), which integrates multiple traditional Chinese medicine databases (including SymMap, TCMID, TCMSP, and TCM-ID) and contains the most comprehensive traditional Chinese medicine and chemical components [13]. Curcumin was also searched in the PubChem database (https://pubchem.ncbi.nlm.nih.gov/) to obtain its corresponding 3D structure and on the simplified molecular input line entry search (SMILES). The SMILES notation was entered into the SwissTargetPrediction database (http://www.swisstargetprediction.ch/) [14] to obtain curcumin’s predicted protein targets and their gene names, which were further screened for a >0.1 probability. The target proteins obtained from the two databases were combined and de-duplicated, and were used as curcumin’s potential targets.

### IS disease target screening

The occurrence and development of IS involve the co-regulation of multiple genes. IS’s targets were searched using “ischemic stroke” as the keyword in four databases: DrugBank (https://www.drugbank.ca/) [15], GeneCards (https://www.genecards.org/) [16], DisGeNET (https://www.disgenet.org/) [17], and the Therapeutic Target Database (TTD) (http://db.idrblab.net/ttd/) [18]. UniProt (https://www.uniprot.org/) [19] was used to convert each database’s target information into standardized gene names, which were summarized and de-duplicated.

### Getting common targets

Curcumin’s potential targets and IS disease targets were imported into the drawing tool InteractiVenn (http://www.interactivenn.net/) [20]. Venn diagrams were drawn online to identify the common targets.

### GO and KEGG enrichment analysis

The common targets were entered into Metascape (https://metascape.org∥) [21]. “Homo sapiens” was selected to conduct the GO and KEGG enrichment analyses to predict the pathways of action and function distribution of the common targets. The GO functional enrichment analysis included biological process, molecular function, and cellular component-related processes. Using the log10(P) as the sorting standard, the 20 most enriched KEGG pathways and their associated top 10 GO functions were selected. Bioinformatics online tools (http://www.bioinformatics.com.cn) were used for visual analysis.

### Construction of a protein–protein interaction network and Curcumin–target–pathway network

The STRING database (https://string-db.org/) [22] can search online for protein-protein interaction (PPI) relationships for protein targets. The curcumin–IS common targets were entered into the STRING database. To obtain the network diagram of the interaction between curcumin’s potential targets and IS’s target proteins, terms such as “Homo sapiens,” “>0.9,” and “hide disconnected nodes in the network” were filtered. The results were exported in a “TSV”-formatted file. The TSV file was imported into Cytoscape 3.9.0 software [23]. We conducted network topology analysis, calculated attribute values such as node degree, combined scores, etc., and drew the network diagram of component–disease common target interaction. The PPI was adjusted according to the node degree and combined scores. The intersection targets were sorted according to node degree value, and the top 10 were selected as the candidate key targets for curcumin’s effects in IS treatment.

The 20 selected KEGG pathway and their enriched target genes were sorted. The files were converted into Network.xlsx and Type.xlsx documents, which were then imported into Cytoscape 3.9.0 software to make a curcumin–target–pathway network diagram. The node degree values of the network were computed and the top 10 with higher values were chosen as the potential primary targets for the curcumin treatment of IS. The final key targets were identified from the PPI network topology analysis and curcumin–target–pathway network diagram.

### Molecular docking

The PDB database (https://www.rcsb.org/) was used to download the 3D crystal structures of key targets, which were then imported into Pymol 2.1 to remove water molecules and residue ligands and AutoDock Tools-1.5.6 to add hydrogen atoms. The 3D structure of curcumin obtained from the PubChem database was imported into Chem3D for energy optimization, and into AutoDock Tools-1.5.6 for hydrogen addition, atom type assignment, etc. The center of the grid box is established based on the interaction between the processed compound (ligand) and the target proteins (receptor) after their importation into AutoDockTools-1.5.6 [24]. Finally, molecular docking was performed by AutoDock software, and we selected the docking conformation with the lowest binding energy. Visualization was done with Pymol 2.1 software and Schrödinger maestro 2018.

### Molecular dynamics

To evaluate the dynamic characteristics and stability of the compound–target protein complex, Gromacs 2020 software was used to run 100 ns MD simulations. In this investigation, the complex generated from the molecular docking results served as the starting conformation. Small molecule ligands were treated with the general force field settings; proteins were treated using AMBER99SB-ILDN force field parameters. Select a periodic stereo box where the atoms of the complex are at least 1.0 nm from the edge of the water box. Select the TIP3P-dominating water model and include salt or chloride ions to balance the charge of the simulated system. The maximum rate of the descent approach and the conjugate gradient method was used to minimize the energy of the solvated system. The system was gradually heated to 300 K in 50 ps and then equilibrated for 50 ps under the NPT (constant number of particles, pressure, and temperature) ensemble. Finally, MD simulations for each equilibrium system were run for 100 ns. The root mean square deviation (RMSD), root mean square fluctuation (RMSF), Hydrogen bond number, and the radius of gyration (RG) were performed on the trajectory data, which were stored every 10 ps.

The binding free energy of the complex was calculated using the MM/GBSA method with the g_MMPBSA script in Gromacs 2020. The binding free energy can be decomposed into molecular mechanical energy and solvation energy. Molecular mechanical energy comprises electrostatic and van der Waal’s interactions, whereas solvation energy comprises polar and nonpolar solvation-free energies [25, 26].

## Results

### Common targets of curcumin and IS

**Fig 2:**
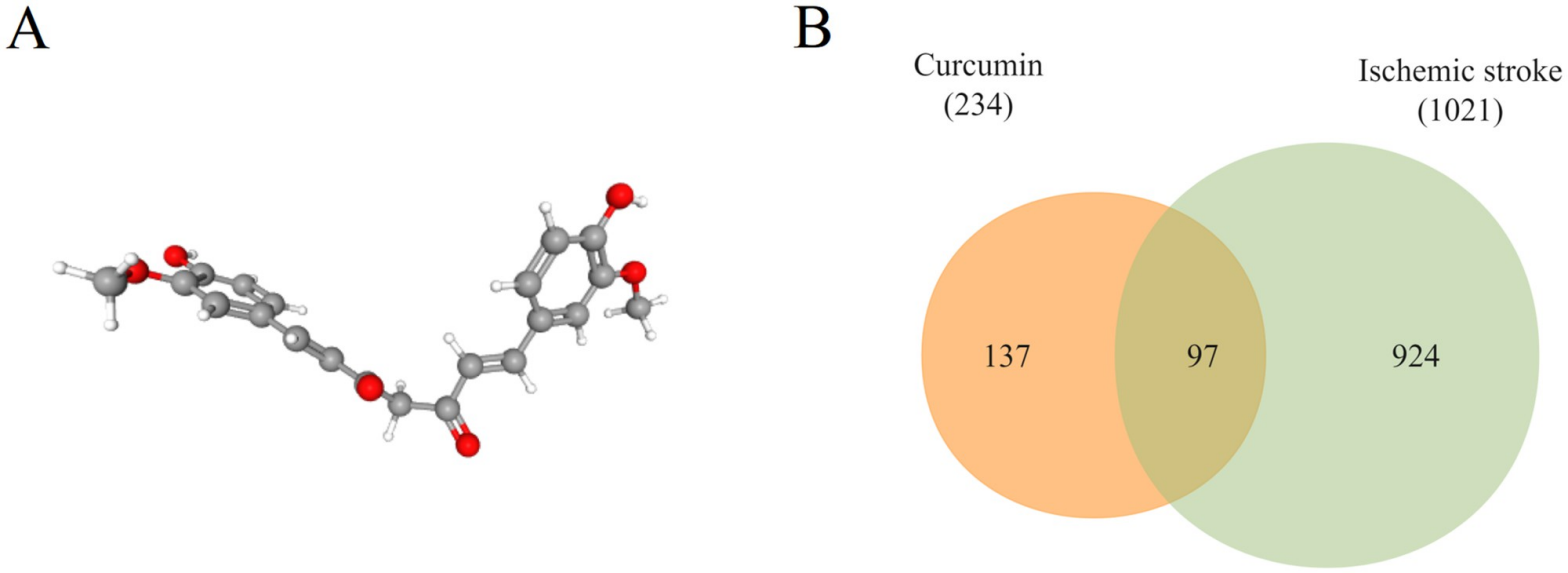
(A) 3D structure of curcumin. (B) Venn diagram of the common target of curcumin and IS.

Curcumin’s 3D structure obtained from PubChem is shown in Fig 2A. The HERB and SwissTargetPrediction databases generated 257 and 64 targets, respectively. Of these, 87 duplicates were removed for a total of 234 potential curcumin targets.

DrugBank, GeneCards (Median Relevance Score ≥ 2), DisGeNET (Relevance Score ≥0.1), and TTD generated 83, 925, 126, and 14 IS disease targets, respectively. During standardization of the target names, 3 targets were removed due to failure to find their control gene names, while 126 duplicate targets were also removed. In total, 1021 IS disease targets were collected.

The Venn diagrams showed that a total of 97 common targets were obtained between curcumin potential targets and IS disease targets (Fig 2B and S1 Table).

### GO and KEGG enrichment analysis

**Fig 3:**
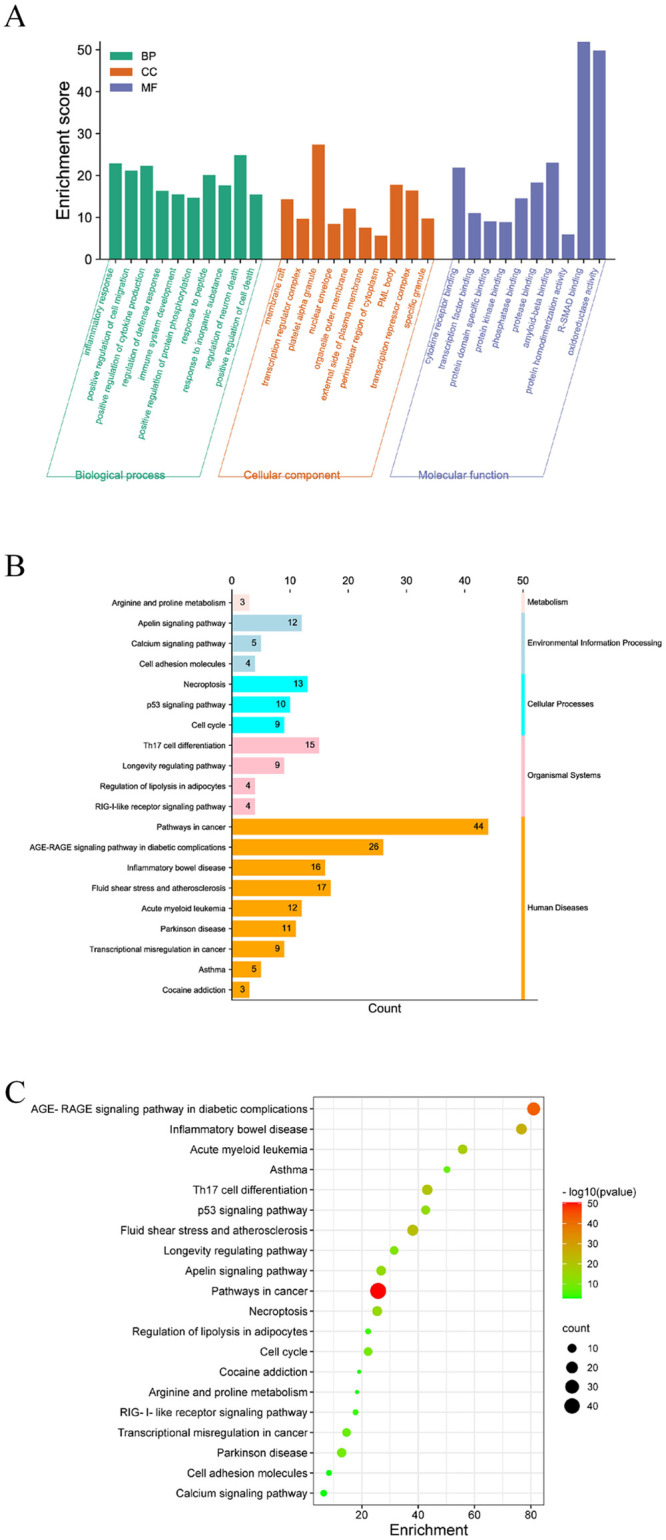
(A) Histogram of GO functional enrichment analysis. (B) Classification diagram for the top 20 KEGG pathways. (C) Bubble Diagram of KEGG enrichment for the top 20 pathways.

GO functional enrichment analysis was performed for the 97 intersection curcumin–IS targets, revealing a total of 1703 biological processes (BP), 120 molecular functions (MF), and 54 cellular components (CC) involved. The top 10 GO enrichment results were visualized and analyzed (Fig 3A). The BP primarily involved neuron death regulation, inflammatory response and positive regulation of cytokine production, cytokine production, and positive regulation of cell migration. The MF primarily involved oxidoreductase activity, acting on single donors with incorporation of molecular oxygen, amyloid-β binding, cytokine receptor binding, and other processes. The CC primarily involved platelet alpha granule, membrane raft, transcription repressor complex, and organelle outer membrane.

**Fig 4:**
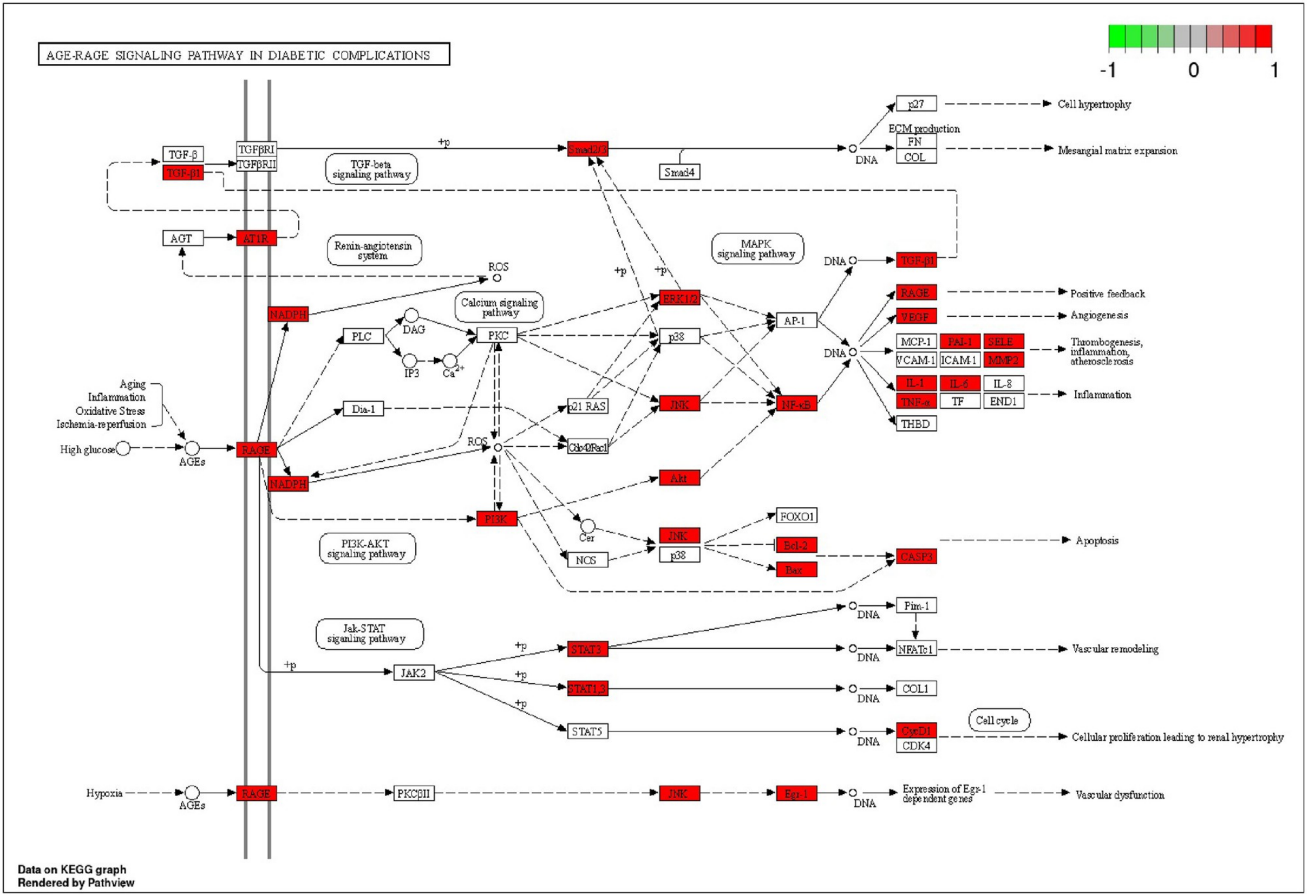
AGE-RAGE signaling pathway in diabetic complications.

The KEGG enrichment results showed that a total of 177 pathways were involved in curcumin treatment of IS. The top 20 pathways can be roughly divided into five categories: human disease, organismal systems, cellular processes, environmental information processing and metabolism (Fig 3B). Curcumin can regulate the AGE-RAGE signaling pathway in diabetic complications, Th17 cell differentiation, the p53 signaling pathway, apelin signaling pathway, necroptosis, and other pathways to treat IS, suggesting that curcumin may affect IS outcomes through intervention of multiple pathways (Fig 3C, S2 Table). Among these, the AGE-RAGE signaling pathway had the highest enrichment, suggesting that it is the most significant pathway involved in curcumin–IS common targets (Fig 4, S2 Table).

### Generation of PPI network, curcumin–target–pathway network and key targets

**Fig 5:**
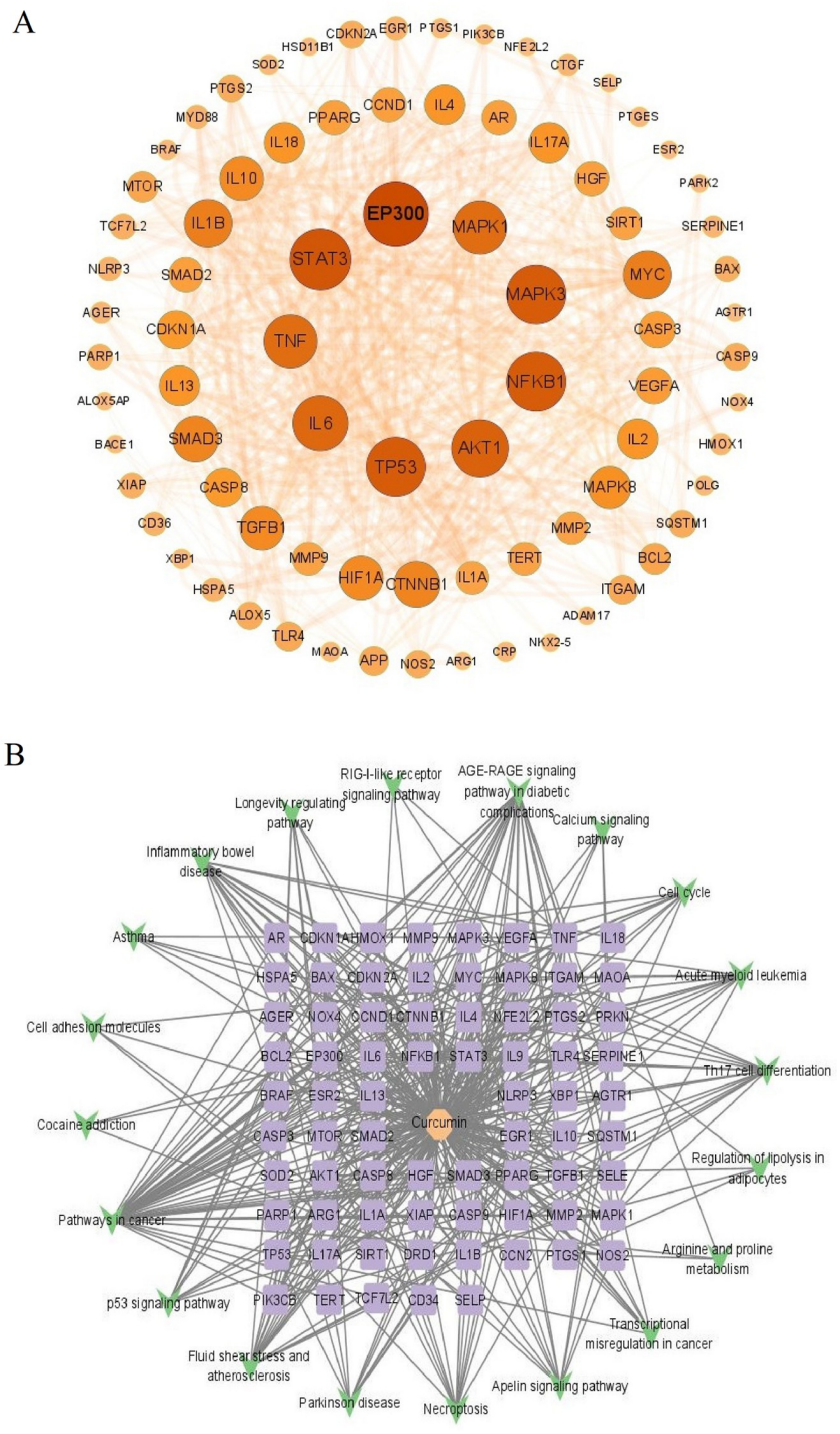
(A) PPI network diagram. (B) Curcumin–pathway–target network diagram. The green quadrilateral represents the KEGG pathway, the purple oval represents the target gene, and the pink hexagon represents curcumin.

Fig 5A shows the PPI network constructed for the 97 common targets using STRING and Cytoscape 3.9.0. Since free targets were hidden during the screening process, the PPI network contained a total of 81 nodes and 688 edges. The average node degree value was 8.494. The size and color of the node reflect the magnitude of degree value; larger and darker nodes indicate a greater target point degree value than smaller and lighter nodes. The thickness and color of the connection between nodes reflect the magnitude of the tightness value; thicker and darker connections indicate greater tightness values. Sorted by node degree value, the top 10 curcumin–IS candidate key targets were EP300, STAT3, MAPK3, TP53, NFKB1, AKT1, IL6, TNF, MAPK1, and IL1B (Table 1).

**Table 1:** The top 10 curcumin–IS candidate key targets according degree.

In PPI network			In Curcumin–pathway–target network		
Target	Protein name	Degree	Target	Protein name	Degree
EP300	Histone acetyltransferase p300	52	NFKB1	Nuclear factor-kappa B subunit 1	20
STAT3	Signal transducer and activator of transcription 3	48	BAX	Apoptosis regulator BAX	14
NFKB1	Nuclear factor-kappa B subunit 1	46	TP53	Cellular tumor antigen p53	14
MAPK3	Mitogen-activated protein kinase 3	46	AKT1	AKT Serine/Threonine Kinase 1	14
TP53	Cellular tumor antigen p53	46	MAPK8	Mitogen-activated protein kinase 8	14
AKT1	AKT Serine/Threonine Kinase 1	44	STATA3	Signal transducer and activator of transcription 3	12
IL6	Interleukin-6	42	SMAD3	Mothers against decapentaplegic homolog 3	12
TNF	Tumor necrosis factor	40	PIK3CB	Phosphatidylinositol 3-kinase catalytic subunit type 3	12
MAPK1	Mitogen-activated protein kinase 1	40	CCND1	G1/S-specific cyclin-D1	12
IL1B	Interleukin-1 beta	34	TNF	Tumor necrosis factor	12

The curcumin–target–pathway network diagram contained 94 nodes, including 73 common targets, 20 KEGG enrichment pathways, and 1 chemical component, curcumin (Fig 5B). The diagram had 462 edges. Ranked by degree value, the top 10 targets were NFKB1, MAPK8, BAX, TP53, AKT1, STAT3, SMAD3, PIK3CB, CCND1, and TNF (Table 1). Among them, NFKB1, TP53, AKT1, STAT3, and TNF also play important roles in the PPI network structure; thus, they are considered the key targets of curcumin for IS treatment.

### Molecular docking results

**Fig 6:**
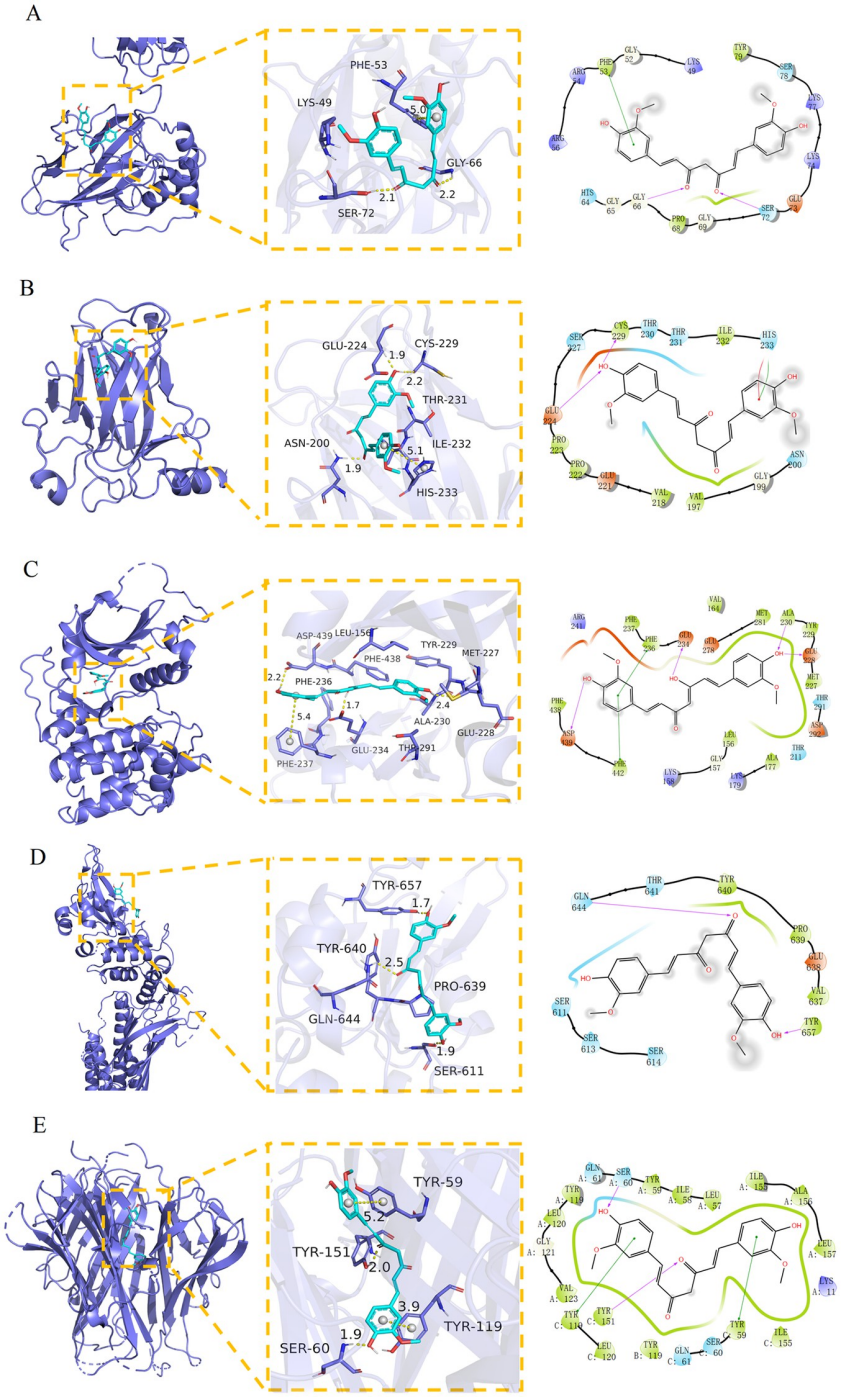
The binding mode of curcumin and 5 Targets. (A) NF-κB–curcumin. (B) TP53–curcumin. (C) AKT1–curcumin. (D) STAT3–curcumin. (E) TNF-curcumin. Yellow dash represents hydrogen bond distance or π-stacking.

The five identified key targets—NFKB1(PDB:2O61), TP53(PDB:5BUA), AKT1(PDB:5BUA), STAT3(PDB:6NUQ), TNF (PDB:7KPA)—and curcumin were used for molecular docking. Five repetitions of molecular docking results showed that the binding energies were each <−5.0 kcal/mol, indicating that curcumin has good binding activity with the five targets (Table 2). The docking score (−10.07 kcal/Mol) of curcumin and TNF was statistically considerably lowest than that of other complexes, indicating that they had the strongest binding activity. The visualization results showed that curcumin primarily relied on hydrogen bonding, π–π conjugation, and water transport interactions to stabilize the docking into the binding pocket of the target protein (Fig 6). Curcumin formed hydrogen bonds with residues SER-72 and GLY-66 in the NF-κB binding site, π–π conjugation with residues PHE-53, and hydrophobic interactions with PHE-53, TYR-79 and PRO-68 (Fig 6A). Curcumin formed hydrogen bonds with residues GLU-224, ASN-200, CYS-229 in TP53 binding site, π–π conjugation with HIS-233, and hydrophobic interactions with CYS-229, ILE-232, and so on (Fig 6B). Curcumin formed strong hydrogen bonding interactions with GLU-234, ALA-230, GLU-228, ASP-439 amino residues on AKT1 protein, π–π conjugation interactions with PHE-442, PHE-236, and hydrophobic interactions with PHE-237, PHE-236, VAL-164 and other residues (Fig 6C). Curcumin formed hydrogen bonds with GLU-644, TYR-657, SER-611 and hydrophobic interactions with residues TYR-740, PRO-639 of STAT3 protein structure (Fig 6D). Curcumin forms strong hydrogen bonding interactions with amino acid residues SER-60 and TYR-151 of the TNF protein structure, π–πconjugation interactions with TYR-59 and TYR-119, and hydrophobic interactions with TYR-151, TYR-119, and other residues (Fig 6E).

**Table 2:** Docking results of curcumin with key target molecules.

Ingredient	Gene	PDB Number	Binding Energy (kcal/mol)		
			Mean	Min	Max
Curcumin	NFKB1	2O61	−6.05±0.38	−6.55	−5.71
	TP53	5BUA	−5.66±0.49	−6.21	−5.11
	AKT1	4GV1	−6.44±0.58	−6.96	−5.59
	STAT3	6NUQ	−5.73±0.50	−6.51	−5.37
	TNF	7KPA	−10.07±0.93*	−11.14	−9.01

* When the docking score was compared, *P*<0.05.

### Molecular dynamics results

**Fig 7:**
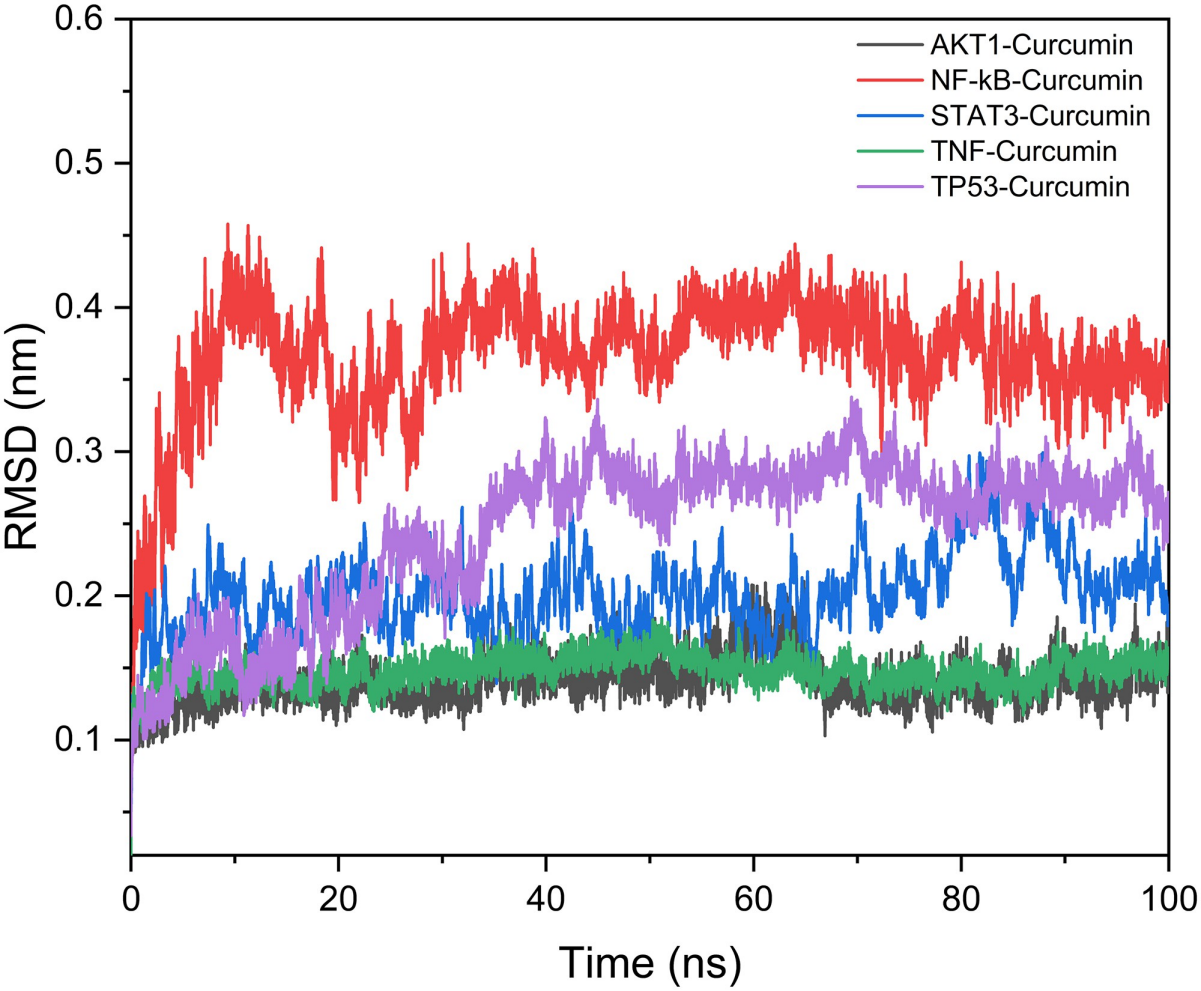
RMSD of the protein−ligand complexs during simulations.

We calculated the RMSD to explain the structural conformations of the complexes and the system’s stability during the simulation. The average RMSD values of curcumin with the key target AKT1, NF–κB, STAT3, TNF, and TP53 were 1.4, 3.7, 2, 1.5, and 2.4 Å, respectively. All molecules had essentially reached dynamic equilibrium with minor fluctuations after 40 ns. Additionally, there were no faults in the RMSD curves. All the above information suggests that the molecules could firmly attach to the protein during the simulation and not dissociate from the protein pocket (Fig 7).

**Fig 8:**
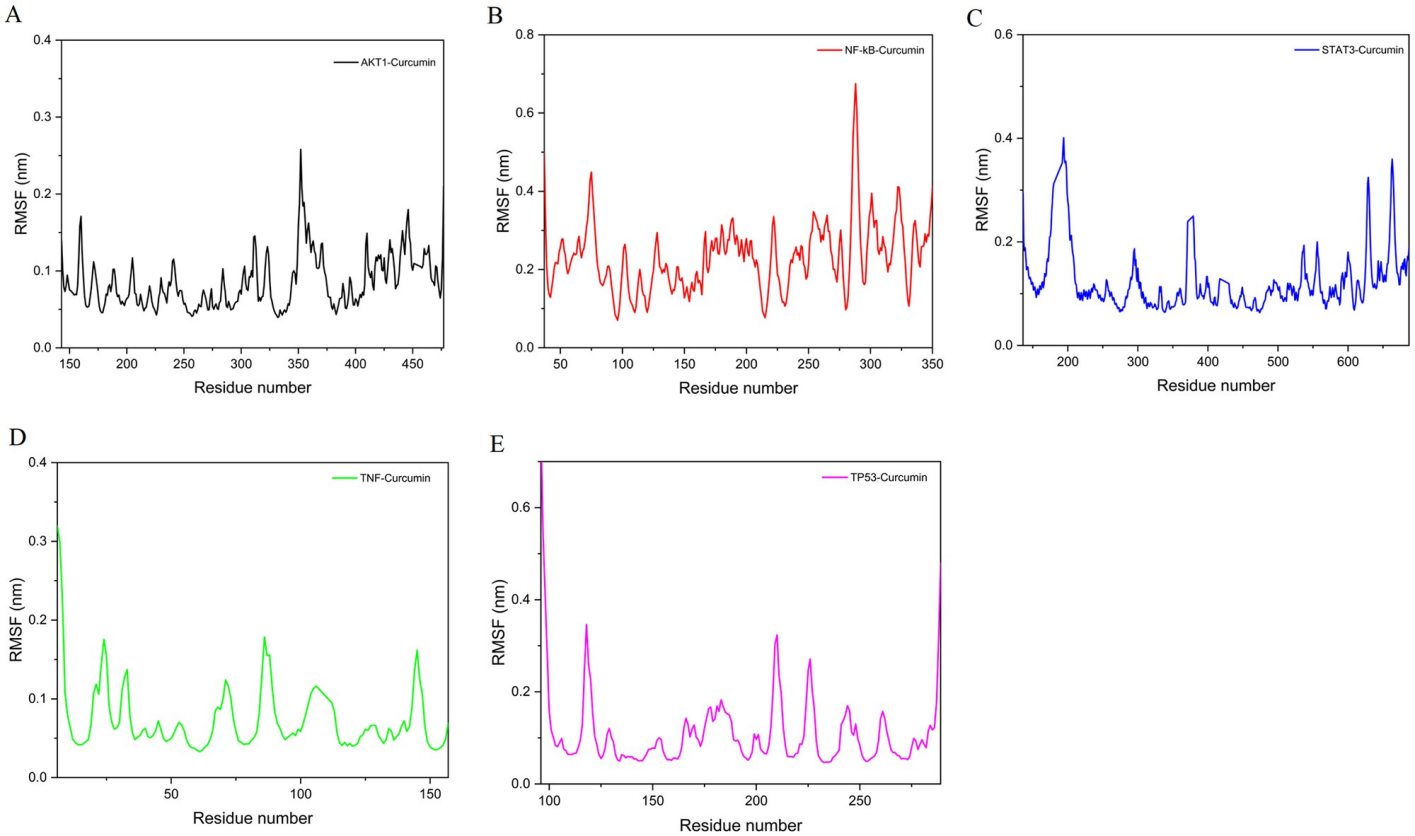
RMSF of the complex. (A) AKT1–curcumin. (B) NF-κB–curcumin. (C) STAT3–curcumin. (D) TNF–curcumin. (E) TP53–curcumin.

The fluctuation of amino acid residues in a protein after small molecule binding is reflected by RMSF. The results show that most amino acid conformation changes during the simulation are minor. Only a few residues underwent large conformational changes, most likely due to their location in the protein’s hinge region. Moreover, TNF–curcumin and AKT1–curcumin had RMSF values less than 2.5 Å (Fig 8).

**Fig 9:**
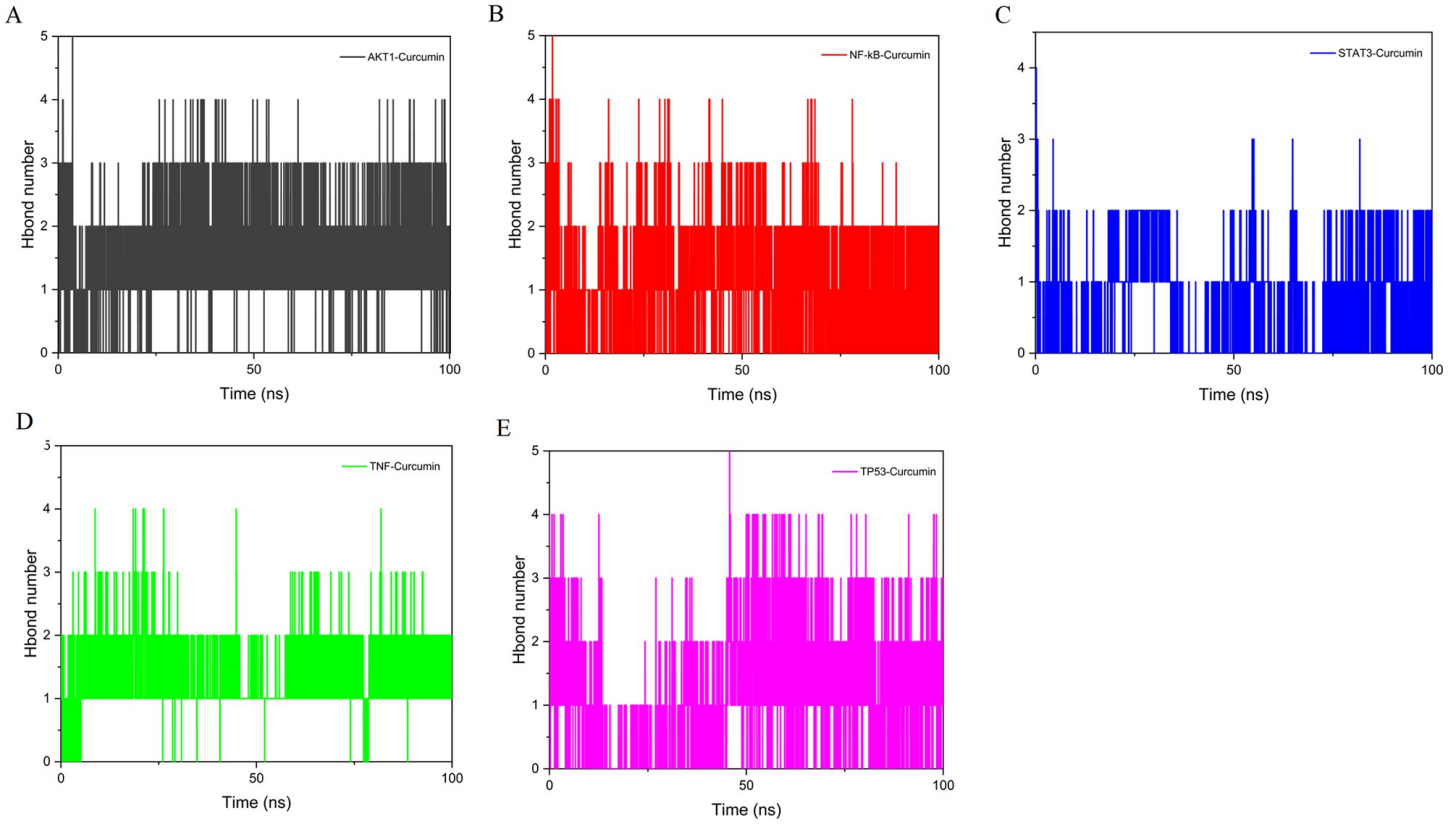
Hydrogen bond number of complex. (A) AKT1–curcumin. (B) NF-κB–curcumin. (C) STAT3–curcumin. (D) TNF–curcumin. (E) TP53–curcumin.

The number of hydrogen bonds in a complex can reflect its binding strength. The ligands and residues in all five protein pockets formed one or more hydrogen bonding interactions. AKT1–curcumin had the highest hydrogen bond density and size, followed by TP53–curcumin, TNF–curcumin, NF-κB–curcumin, and STAT3–curcumin (Fig 9).

**Fig 10:**
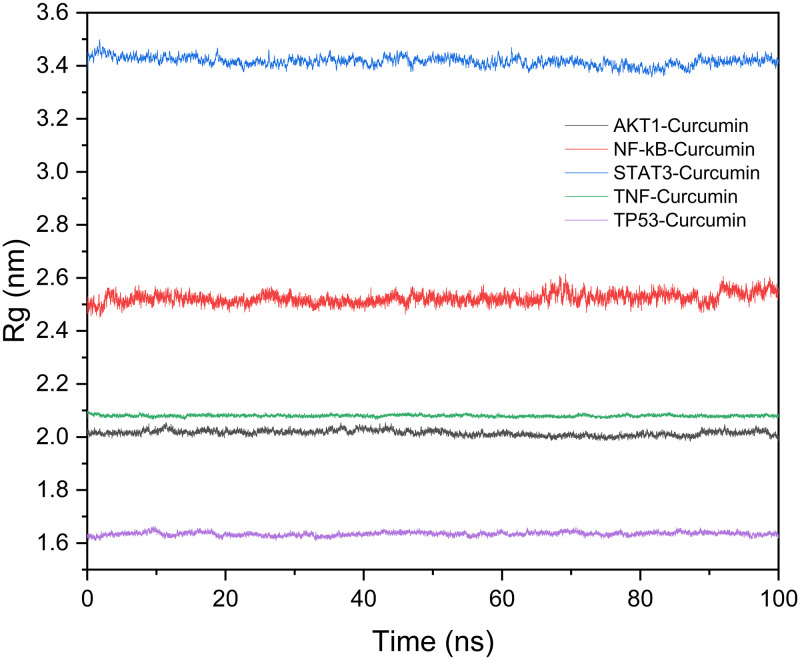
Compactness of the protein according to Rg.

Rg evaluated the compactness of the protein structures. In descending order of compactness, the complexes are TP53–curcumin, AKT1–curcumin, TNF–Curcumin, NF-κB–Curcumin, and STAT3–curcumin (Fig 10).

The binding free energy can be used to determine the change in binding pattern and stability of the ligands and proteins. The free energy formed by the binding of AKT1, NF-κB, STAT3, TNF, and TP53 to curcumin was −94.891±12.951, −86.48±7.536, −60.019±13.929, -114.599±11.442 and −53.592±18.824 kJ/mol, respectively (Table 3). And TNF–curcumin has the lowest binding free energy and is the most strongly bound complex, which is consistent with the docking results. The main reason is that the active site of TNF is long, narrow, and mainly composed of hydrophobic amino acids. The long-form compound has a better chance of forming a solid bond with the TNF protein pocket, allowing the tiny molecule to bind to the protein. Additionally, the energy decomposition results show that van der Waal’s forces, followed by electrostatic interactions, significantly contribute to the stabilization of small molecules (Table 3). Nonpolar solvation energy is only a minor contributor.

**Table 3:** The binding free energy by MMGBSA (kJ/mol).

Name	ΔEvdw	ΔEelec	ΔGgb	ΔGsa	ΔGbinding
AKT1	−198.682±9.012	−4.244±17.869	127.613±13.721	−19.577±0.701	−94.891±12.951
NF−κB	−140.857±5.517	−30.322±7.597	99.705±3.394	−15.007±0.619	−86.48±7.536
STAT3	−106.719±11.833	−19.556±10.442	78.95±7.154	−12.693±0.74	−60.019±13.929
TNF	−217.975±10.204	−35.831±7.754	161.864±12.65	−22.656±0.811	−114.599±11.442
TP53	−108.844±18.118	−42.861±7.841	110.909±17.786	−12.796±1.595	−53.592±18.824

ΔEvdw = van der Waals energy; ΔEelec = eletrostatic energy; ΔGgb = eletrostatic contribution to solvation; ΔGsa = non-polar contribution to solvation; ΔGbinding = the bonding free energy.

## Discussion

Cranial injury in patients with IS is caused by various pathological processes, such as inflammatory response, oxidative stress, platelet activation, and blood-brain barrier disruption [27]. Curcumin has multifunctional characteristics, which can reduce nerve tissue damage through multiple target effects with few side effects [28]. This study systematically investigated the potential targets and molecular mechanisms of curcumin on IS.

The results showed that curcumin and IS had 97 common targets. The KEGG enrichment analysis of common targets indicated that curcumin might play a role in IS through regulation of the AGE-RAGE signaling pathway, Th17 cell differentiation, the p53 signaling pathway, the apelin signaling pathway, necrotic apoptosis, and other pathways. Among these, the AGE-RAGE pathway was the most enriched, indicating that it may be the core pathway in curcumin–IS effects. AGE-RAGE plays an important role in diabetic complications, and stroke has been identified as one of the major vascular complications of diabetes [29]. AGE is a harmful molecule formed by macromolecular glycosylation, and RAGE is one of its transmembrane receptors [30]. AGE-RAGE can activate NFKB1 through effects on the MAPK, PI3K/Akt, JAK/STAT, and Nox/ROS signaling pathways, as well as others. The activated NFKB1 enters the nucleus to promote expression of TNF, IL-6, and other inflammatory factors and oxidative stress-related molecules. Ultimately, NFKB1 increases inflammation in the injured neurons and induces a continuous oxidative stress reaction which damages the vascular endothelium, thus further increasing IS severity [31–34]. The inflammatory reaction may, in turn, increase plasma AGE levels and activate the AGE-RAGE pathway to form a continuous cycle of damage [35]. Although AGE-RAGE was the most enriched pathway, Th17 cell differentiation, the p53 signaling pathway, the apelin signaling pathway, and necrotic apoptosis also contribute to inflammation, which may be mediated by curcumin following IS. Curcumin may also improve brain injury outcomes by regulating Th17 cell differentiation. T cells, a harmful component of the IS response, enter the ischemic injury area 24 hours after stroke onset. Th17 is a subtype of T lymphocytes [36] which can secrete IL-17, a pro-inflammatory factor. IL-17 plays a key role in the inflammation caused by ischemic brain injury [37]. The p53 signaling pathway can interact with Bcl.2 family multi-domain members and directly participate in the endogenous apoptosis pathway [38]. Xie et al. demonstrated for the first time that curcumin could increase Bcl-2 expression and inhibit Bax activation, promoting neuronal survival and exerting antiapoptotic biological activity [39]. P53 channels are a promising therapeutic target to reduce stroke injury; they can induce a strong neuroprotective effect against cerebral ischemia-reperfusion injury and significantly reduce brain injury [40]. The apelin signaling pathway has neuroprotective effects against aspartate-mediated excitotoxicity and can reduce inflammatory factor levels, especially TNF expression levels [41]. Necroptosis is one mechanism of neuronal death following stroke. Necrosis causes cell lysis, which releases potential immune inflammatory cytokines to induce a strong neuroinflammatory response, thereby aggravating brain tissue injury from cerebral ischemia processes and cerebral ischemia-reperfusion [42].

The results of the PPI and component–target–pathway network using network pharmacology showed that NFKB1, TP53, AKT1, STAT3, and TNF were key targets in IS regulated by curcumin. NFKB1 is a key transcription factor in the NF-κB signaling pathway and is involved in the release of pro-inflammatory factors such as TNF-α, IL-6, IL-1β, and NLRP3. Curcumin’s anti-inflammatory effects are closely related to its inhibition of the NF-κB signaling pathway [43]. TP53, as a tumor suppressor, can activate pro-apoptotic factors and participate in the apoptotic process, thus further increasing IS severity [44]. AKT1 is highly expressed in the nerve cytoplasm and is a key growth factor, inducing the survival of neurons affected by stroke [45]. AKT1 activation can initiate a downstream cascade reaction of the PI3K/Akt signaling pathway, and it further phosphorylates a series of downstream substrates such as Bad, Caspase-3, and GSK-3β, thereby promoting cell survival and anti-apoptosis [46]. Some studies have suggested that STAT3 activation can not only enhance the microglia’s anti-inflammatory response and inhibit their pro-inflammatory response, but that it can also promote endogenous angiogenesis and neurogenesis in an ischemic brain to effectively inhibit neuronal apoptosis [47, 48]. At present, experiments have demonstrated that curcumin can improve neuronal survival rate by activating JAK2/STAT3 signals, as well as reduce cerebral ischemia-reperfusion injury [49]. Some studies suggest that STAT3 activation might cause neuronal damage [50]. Microglia are resident macrophages of the central nervous system and are known to be involved in the maintenance of the central nervous system stability under normal conditions. They can be roughly divided into pro-inflammatory (M1) and anti-inflammatory phenotypes (M2) [51, 52]. Following ischemia, M1 are rapidly activated and release a variety of inflammatory mediators, such as IL-1 β, IL6, and TNF [53]. Pro-inflammatory factors lead to an inflammatory cascade reaction, aggravate inflammatory expression in the injured area, and participate in a variety of stroke-induced pathophysiological changes, with persistent inflammation causing irreversible damage to the brain cells [54]. At present, studies have shown that curcumin indirectly promotes functional recovery by regulating the polarization of pro-inflammatory microglia/macrophages to anti-inflammatory ones; reducing the release of pro-inflammatory factors such as IL-1, IL-6, and TNF; and reducing inflammatory response [55].

We used molecular docking to predict the binding conformation and intermolecular affinities of the five key targets and compounds. The docking results showed that curcumin can more firmly bind to key proteins through hydrogen bonds, π–π conjugation, and hydrophobic interactions, indicating that curcumin can be used for the treatment of IS by interacting with multiple targets. In this study, the Autodock program was selected for molecular docking because it is now a commonly used tool that can accurately predict the conformation of small molecule ligands within the predicted target binding region [24, 56]. When a protein recognizes or binds a ligand, its conformation changes, but molecular docking ignores the flexibility of this target binding site [57]. This limitation can be overcome by molecular docking.

MD can identify the trajectory and temporal changes of the complex, as well as discover atomic interactions between ligands and protein amino acid residues, which can validate and supplement the results of molecular docking [58]. In this study, the analysis of various data based on MD simulation trajectories shows that curcumin has a strong affinity for the five proteins, which promotes the formation of stable complexes between small molecules and proteins, thereby exerting curcumin’s active role. The insufficient collection of molecular conformations and the considerable processing cost necessary for simulations should not be neglected [59].

Numerous biological properties of curcumin have been reported, including antioxidant, anti-inflammatory, antibacterial, and neuroprotective effects [11, 60]. Our study revealed the pharmacological mechanism of curcumin multipotency in IS. However, there are still some challenges in its therapeutic application. Curcumin’s limited solubility in water, low bioavailability, and brief half-life have prevented it from reaching its full potential for clinical use [61]. Drug delivery systems such as nanoparticles, hydrogels, and other carriers provide solutions [62]. However, the synthesis protocols of curcumin and delivery systems should still be optimized to ensure safety, efficacy, and stability [62, 63].

## Conclusions

In conclusion, this study used network pharmacology to identify and analyze potentially relevant curcumin mechanisms for the treatment of IS. The results showed that, in the context of IS-related processes, curcumin may exert anti-inflammatory and apoptosis-inhibiting pharmacological effects through multiple targets—NFKB1, TP53, AKT1, STAT3, and TNF—and by regulating multiple pathways such as the AGE-RAGE signal pathway, Th17 cell differentiation, the p53 signal pathway, the apelin signal pathway, and necrotic apoptosis. Of these, the AGE-RAGE signal pathway was the most enriched pathway of the common curcumin–IS targets, indicating that it may have a critical role in IS treatment. However, the complex development of diseases and pharmacodynamic processes of bioactive ingredients are dynamic, whereas computer biotechnology is a static network analysis, has limited simulation time, etc. Therefore, further experiments and clinical trials are still needed for validation. In the future, dynamic network data analysis or simulation with sufficient time can be developed to drive the data results as close to the actual results as possible, minimizing needless trial-and-error experiments and assisting in the acceleration of the clinical translation of drugs.

## Supporting information

S1 Table The common targets of curcumin and IS. (DOC)

S2 Table The top 20 pathways of KEGG enrichment analysis. (DOC)
