# Supplementary material for: Curcumin’s mechanism of action against ischemic stroke: A network pharmacology and molecular dynamics study
Source: PLoS One. 2023 Jan 4;18(1):e0280112. doi: 10.1371/journal.pone.0280112 (PMC9812305; doi:10.1371/journal.pone.0280112)
Supplement: S1 Table — (DOC) [file pone.0280112.s001.doc]

| Number | Target | Number | Target | Number | Target | Number | Target | Number | Target |
| --- | --- | --- | --- | --- | --- | --- | --- | --- | --- |
| 1 | ALOX5AP | 21 | SMAD2 | 41 | CDKN2A | 61 | ITGAM | 81 | PRKN |
| 2 | CRP | 22 | AKT1 | 42 | AGER | 62 | STAT3 | 82 | EGR1 |
| 3 | TNF | 23 | SIRT1 | 43 | CD36 | 63 | CHAT | 83 | SQSTM1 |
| 4 | IL6 | 24 | PPARG | 44 | CCND1 | 64 | MMP8 | 84 | CASP8 |
| 5 | AGTR1 | 25 | PTGS2 | 45 | NLRP3 | 65 | MTOR | 85 | NFKB1 |
| 6 | SERPINE1 | 26 | NFE2L2 | 46 | CD34 | 66 | SST | 86 | NOX4 |
| 7 | MMP9 | 27 | IL4 | 47 | TCF7L2 | 67 | XIAP | 87 | BAX |
| 8 | SELP | 28 | MMP2 | 48 | IL17A | 68 | ARG1 | 88 | MYC |
| 9 | IL1B | 29 | MAPK3 | 49 | XBP1 | 69 | MYD88 | 89 | AKR1B1 |
| 10 | APP | 30 | NKX2-5 | 50 | CX3CR1 | 70 | CCR6 | 90 | ABCC1 |
| 11 | VEGFA | 31 | MIR155 | 51 | SERPINA1 | 71 | DRD1 | 91 | MAOA |
| 12 | TLR4 | 32 | BACE1 | 52 | IL2 | 72 | TERT | 92 | PTGES |
| 13 | TP53 | 33 | TGFB1 | 53 | PARP1 | 73 | IL9 | 93 | EP300 |
| 14 | IL10 | 34 | CASP9 | 54 | HSPA5 | 74 | CCN2 | 94 | HSD11B1 |
| 15 | CASP3 | 35 | HMOX1 | 55 | BRAF | 75 | CD68 | 95 | MMP13 |
| 16 | SOD2 | 36 | NOS2 | 56 | IL13 | 76 | ADAM17 | 96 | CDKN1A |
| 17 | HIF1A | 37 | IL18 | 57 | BCL2 | 77 | MAPK8 | 97 | PIK3CB |
| 18 | ALOX5 | 38 | IL1A | 58 | CTNNB1 | 78 | ESR2 |  |  |
| 19 | SELE | 39 | PTGS1 | 59 | MAPK1 | 79 | AR |  |  |
| 20 | SMAD3 | 40 | POLG | 60 | TREM2 | 80 | HGF |  |  |
