# Supplementary material for: Curcumin’s mechanism of action against ischemic stroke: A network pharmacology and molecular dynamics study
Source: PLoS One. 2023 Jan 4;18(1):e0280112. doi: 10.1371/journal.pone.0280112 (PMC9812305; doi:10.1371/journal.pone.0280112)
Supplement: S2 Table — (DOC) [file pone.0280112.s002.doc]

| **Pathway** | **-Log10(P)** | **Enrichment** | **Count** | **Tagets** |
| --- | --- | --- | --- | --- |
| Pathways in cancer | 50.44409296 | 25.80783195 | 44 | AGTR1, AKT1, XIAP, AR, BAX, CCND1, BCL2, BRAF, CASP3, CASP8, CASP9, CDKN1A, CDKN2A, CTNNB1, EP300, ESR2, MTOR, HGF, HIF1A, HMOX1, IL2, IL4, IL6, IL13, SMAD2, SMAD3, MMP2, MMP9, MYC, NFE2L2, NFKB1, NOS2, PIK3CB, PPARG, MAPK1, MAPK3, MAPK8, PTGS2, STAT3, TCF7L2, TERT, TGFB1, TP53, VEGFA |
| AGE-RAGE signaling pathway in diabetic complications | 42.65425907 | 80.97793814 | 26 | AGER, AGTR1, AKT1, BAX, CCND1, BCL2, CASP3, EGR1, IL1A, IL1B, IL6, SMAD2, SMAD3, MMP2, NFKB1, SERPINE1, PIK3CB, MAPK1, MAPK3, MAPK8, SELE, STAT3, TGFB1, TNF, VEGFA, NOX4 |
| Inflammatory bowel disease | 25.7032059 | 76.66550357 | 16 | IL1A, IL1B, IL2, IL4, IL6, IL10, IL13, IL17A, IL18, SMAD2, SMAD3, NFKB1, STAT3, TGFB1, TLR4, TNF |
| Fluid shear stress and atherosclerosis | 21.72800146 | 38.09144849 | 17 | AKT1, BCL2, CTNNB1, HMOX1, IL1A, IL1B, MMP2, MMP9, NFE2L2, NFKB1, PIK3CB, MAPK8, SELE, TNF, TP53, VEGFA, SQSTM1 |
| Th17 cell differentiation | 20.05555725 | 43.25744559 | 15 | MTOR, HIF1A, IL1B, IL2, IL4, IL6, IL17A, SMAD2, SMAD3, NFKB1, MAPK1, MAPK3, MAPK8, STAT3, TGFB1 |
| Acute myeloid leukemia | 17.51192763 | 55.78273581 | 12 | AKT1, CCND1, BRAF, MTOR, ITGAM, MYC, NFKB1, PIK3CB, MAPK1, MAPK3, STAT3, TCF7L2 |
| Necroptosis | 14.33744372 | 25.46476042 | 13 | PARP1, XIAP, BAX, BCL2, CASP8, IL1A, IL1B, MAPK8, STAT3, TLR4, TNF, SQSTM1, NLRP3 |
| Apelin signaling pathway | 13.54762218 | 26.88808129 | 12 | AGTR1, AKT1, CCND1, CCN2, EGR1, MTOR, SMAD2, SMAD3, NOS2, SERPINE1, MAPK1, MAPK3 |
| p53 signaling pathway | 13.42012817 | 42.66487784 | 10 | BAX, CCND1, BCL2, CASP3, CASP8, CASP9, CDKN1A, CDKN2A, SERPINE1, TP53 |
| Longevity regulating pathway | 10.89416743 | 31.4953087 | 9 | AKT1, BAX, MTOR, NFKB1, PIK3CB, PPARG, SOD2, TP53, SIRT1 |
| Cell cycle | 9.522975672 | 22.2466863 | 9 | CCND1, CDKN1A, CDKN2A, EP300, SMAD2, SMAD3, MYC, TGFB1, TP53 |
| Parkinson disease | 8.990536409 | 12.87966049 | 11 | BAX, CASP3, CASP9, DRD1, HSPA5, MAOA, NFE2L2, PRKN, MAPK8, TP53, XBP1 |
| Transcriptional misregulation in cancer | 7.907579042 | 14.59938789 | 9 | BAX, CDKN1A, IL6, ITGAM, MMP9, MYC, NFKB1, PPARG, TP53 |
| Asthma | 7.310762907 | 50.23445294 | 5 | IL4, IL9, IL10, IL13, TNF |
| Regulation of lipolysis in adipocytes | 4.491253363 | 22.2466863 | 4 | AKT1, PIK3CB, PTGS1, PTGS2 |
| RIG-I-like receptor signaling pathway | 4.108868847 | 17.79734904 | 4 | CASP8, NFKB1, MAPK8, TNF |
| Cocaine addiction | 3.274842438 | 19.06858826 | 3 | DRD1, MAOA, NFKB1 |
| Arginine and proline metabolism | 3.223658401 | 18.32080049 | 3 | ARG1, MAOA, NOS2 |
| Calcium signaling pathway | 2.966547771 | 6.488616838 | 5 | AGTR1, DRD1, HGF, NOS2, VEGFA |
| Cell adhesion molecules | 2.860186556 | 8.361170691 | 4 | CD34, ITGAM, SELE, SELP |
